# RETRACTED: Elshayb et al. Green Synthesis of Zinc Oxide Nanoparticles: Fortification for Rice Grain Yield and Nutrients Uptake Enhancement. *Molecules* 2021, *26*, 584

**DOI:** 10.3390/molecules31091493

**Published:** 2026-04-30

**Authors:** Omnia M. Elshayb, Khaled Y. Farroh, Heba E. Amin, Ayman M. Atta

**Affiliations:** 1 Rice Research and Training Center, Field Crops Research Institute, Agricultural Research Center Sakha, P.O. Box, Kafrelsheikh 33717, Egypt; 2 Nanotechnology and Advanced Materials Central Lab., Agricultural Research Center, P.O. Box, Giza 588, Egypt; khaledfarroh@yahoo.com; 3 Department of Food Industry, Faculty of Agriculture, Mansoura University, Mansoura 35516, Egypt; heba_emad1127@hotmail.com; 4 Chemistry Department, College of Science, King Saud University, Riyadh 11451, Saudi Arabia

The journal retracts the article titled “Green Synthesis of Zinc Oxide Nanoparticles: Fortification for Rice Grain Yield and Nutrients Uptake Enhancement” [1], cited above.

Following publication, concerns were brought to the attention of the Editorial Office regarding the integrity of a number of images presented in this publication [1].

In accordance with standard journal procedures, the Editorial Office and Editorial Board conducted an investigation that confirmed a duplication between Figure 2D in [1] and Figure 2(b1) from a previously published article [2], produced by a different authorship group, and presenting different experimental conditions. Although the authors cooperated with the investigation, the explanations and supporting materials provided were not deemed sufficient by the Editorial Board to resolve the identified concerns. As a consequence, the Editorial Board has lost confidence in the reliability of the reported findings and has decided to retract this publication in accordance with MDPI’s retraction policy.

This retraction was approved by the Editor-in-Chief of *Molecules* Journal.

The authors have been informed of this retraction but did not provide a comment on this decision.

## References

[1] Elshayb O.M., Farroh K.Y., Amin H.E., Atta A.M. (2021). RETRACTED: Green Synthesis of Zinc Oxide Nanoparticles: Fortification for Rice Grain Yield and Nutrients Uptake Enhancement. Molecules.

[2] Abu-Taweel G.M., Attia M.F., Hussein J., Mekawi E.M., Galal H.M., Ahmed E.I., Allam A.A., El-Naggar M.E. (2020). Curcumin nanoparticles have potential antioxidant effect and restore tetrahydrobiopterin levels in experimental diabetes. Biomed. Pharmacother..

